# Surviving the Holocaust: Socio-demographic Differences Among Amsterdam Jews

**DOI:** 10.1007/s10680-016-9403-3

**Published:** 2017-01-23

**Authors:** Peter Tammes

**Affiliations:** Centre for Academic Primary Care (CAPC), School of Social and Community Medicine (SSCM), University of Bristol, Canynge Hall, 39 Whatley Road, Bristol, BS8 2PS UK

**Keywords:** Holocaust, The Netherlands, Demography, Survival, Mortality, Jewish population

## Abstract

This study determined the victimisation rate among Amsterdam Jews and socio-demographic differences in surviving the Holocaust. After linking a registration list of over 77,000 Jewish inhabitants in 1941 to post-war lists of Jewish victims and survivors, the victimisation rate lies between 74.3 and 75.3 %. Differences in survival chances and risk of being killed are examined by using multivariable logistic and Cox regression analyses. While male Jews had a reduced risk of death, in the end their survival chances hardly differed from females. Though Jews aged 6–14 and 31–50 initially had a lower risk of death, in the end compared with Jews aged 15–30 they had lower survival chances, just as Jews aged 50+. For Jews aged 0–5, it was the other way around. Immigrants showed better survival chances than native Jews. German Jews showed better survival chances than Dutch Jews, but Polish and other Jewish nationals showed highest survival chances. Jews who had abandoned Judaism had better survival chances than Jews belonging to an Israelite congregation. Divorced, widowed and unmarried adult Jews had better survival chances than married Jews and their children; Jews married to non-Jews, however, had one of the highest survival chances. Jews in the two highest social classes had better survival chances than jobless Jews. These findings indicate that survival was not random but related to socio-demographic characteristics. This sheds light on demographic consequences of conflict and violence: Nazi persecution reduced the Amsterdam Jewish community drastically, and socio-demographic differences in survival impacted the post-war Jewish population structure.

**Electronic supplementary material:**

The online version of this article (doi:10.1007/s10680-016-9403-3) contains supplementary material, which is available to authorized users.

## Introduction

Differences in Jewish victimisation rates, i.e. proportions of Jews being directly or indirectly killed by Nazi Germany, among occupied states and regions (e.g. Fein 1979; Benz 1991) and Dutch municipalities (Croes and Tammes 2006) are well-known. Investigations into differences in individual survival chances are scarce but are getting the attention of social scientists and social historians (e.g. Anders and Dubrovskis 2003; Mercklé and Zalc 2014; Zalc et al. 2012). Some scholars such as Van Imhoff et al. (2001) and Presser (1965, part 2: 509–510) are of the opinion that the exact demographic story of the destruction of Dutch Jewry during the Nazi period will quite likely never be written. So far, scholars have used aggregated data (e.g. Van Imhoff et al. 2001) or individual-level data for special groups, e.g. returnees from camps (Van de Vosse 1947) or Jews caught in hiding (Houwink ten Cate 1989) to make some statements about the demographic story of the destruction of Dutch Jewry. This study uses an individual-level approach by investigating the individual fate of more than 77,000 Jews living in Amsterdam in 1941—that is more than half of all Jews in the Netherlands—using a retrieved German Nazi registration list of Jewish inhabitants and post-war lists of victims and survivors. This approach results in a more accurate demographic story of the destruction of Dutch Jewry during the Nazi period.

Studying socio-demographic differences in Amsterdam Jews’ Holocaust survival chances might also contribute to the emerging field of demography of conflict and violence among demographers and peace researchers (Brunborg and Tabeau 2005; Brunborg and Urdal 2005). Following the guidelines of Brunborg and Tabeau (2005) mentioned in a special issue on the demography of conflict and violence in the *European Journal of Population*, the present study can contribute in several ways to this emerging field. First, victims of conflict or violence are not always related to combat—they could also be victims of ethnic cleansing and genocide, or in this case the Holocaust. Second, this study presents a matching procedure to link lists of victims and other deaths to a population list and uses more advanced statistical techniques to investigate survival chances and risk of death due to the Holocaust. Third, studying individual survival chances might expand on what we know about the demographic consequences of conflict and violence for mortality, or in this case socio-demographic differences in Holocaust survival rates.

Some studies into demographic consequences of conflict such as the killings in Srebrenica (Brunborg et al. 2003) and the war-related deaths in Bosnia and Herzegovina (Tabeau and Bijak 2005) used an individual-level approach to identify every victim in order to arrive at a highly reliable estimate of the number of victims and to estimate the probabilities of being a victim. This study on survival among Amsterdam Jews will follow up on those studies by using an individual-level approach to victimisation. Following Brunborg et al. (2003), the first research question of this article is: What is the victimisation rate among Amsterdam Jews? To answer this question, the original registration list of Amsterdam Jews is linked to lists of Holocaust victims and survivors. Following up on Ellis and Rawicki (2014) as to whether Jews survived just by luck or whether survival was selective, the second research question is: Who had higher chances of surviving the Holocaust? To answer this question, this study focuses on some key socio-demographic characteristics such as age, gender, nationality, civil status, social class and religious affiliation. Differences in survival are examined by using multivariable logistic and Cox regression models.

## Previous Findings on Socio-demographic Differences in Jewish Survival

The *American Jewish Yearbook 1948*–*1949* published estimated losses suffered by Jews in 14 occupied countries (Shapiro and Sapir 1948). The proportion of losses for the Netherlands was highest among Western European countries and equalled that of some Eastern European countries. In later studies such as that of Benz (1991), victimisation numbers and rates were more precisely calculated; Hirschfeld (1991) estimated the Dutch victimisation rate at 72.9 % based on the number of deported, returnee and non-deported Jews. Since the persecution of Jews in Eastern Europe differed much in timing and method, the Dutch victimisation rate was especially compared with those of Belgium and France (e.g. Blom 1989), 40 and 25 %, respectively. Though differences and similarities between the Netherlands, Belgium and France in occupation regime, level of anti-Semitism, anti-Jewish regulations and other factors have been studied (e.g. Griffioen and Zeller 2008), differences in socio-demographic composition of Jewish communities in those countries are of more interest to this study.

To compare socio-demographic compositions, the focus is on three cities whose victimisation rate impacted the national rates: Amsterdam in the Netherlands (Croes and Tammes 2006: 39–42), Antwerp in Belgium (Saerens 2000: 745), and Paris in France (Adler 1987: 14, 233). Compared to Amsterdam, the Jewish communities of Antwerp and Paris had a higher proportion of immigrants. In both those cities, Polish Jews formed the biggest immigrant group, whereas in Amsterdam these were German Jews (Adler 1987: 10; Saerens 2000: 20, 648; Veffer 1942: 22–23). Since most Eastern European Jews were more traditional, assimilation tendencies such as secularisation and out-marriage might have been stronger in Amsterdam, while Yiddish was widely used in Antwerp and Paris (Adler 1987: 4; Saerens 2000: 20). In all three cities, a large majority was working in trading or commerce, though in different branches (Adler 1987: 18–19; Saerens 2000: 12; Tammes 2012b). In Antwerp most Jews lived in a district adjacent to the central train station (Saerens 2000: 23), while in both Amsterdam and Paris Jews lived more widespread throughout the city; Jewish immigrants were more concentrated (Tammes 2011; Adler 1987: 5, 10–12). In both Antwerp and Paris, Jewish immigrants were overrepresented among the deported Jews (Saerens 2000: 648; Wetzel 1991: 131, 134), suggesting lower survival rates than native Jews. Differences in survival rates related to other socio-demographic characteristics are unknown, and the impact of socio-demographic characteristics on survival chances is not examined simultaneously.

The Dutch Red Cross [*Nederlandsche Roode Kruis (NRK)*] (Van de Vosse 1947) made a first attempt to show socio-demographic differences in survival of Jews living in the Netherlands during World War II. Van de Vosse (1947) used deportation lists, death registers and a list of survivors to estimate survivor numbers. About 16,000 of the survivors were never deported because they were exempted from deportation or were in hiding. About 5500 deported Jews returned and registered in the Netherlands. Studying the list of returnees, Van de Vosse (1947) concluded that relatively more women had returned, and that nearly all Jews younger than 16 and older than 50 had perished (see also Presser 1965, part 2: 511). The lists of registered returnees, however, are incomplete and the demographic profile of those survivors who were never deported was not investigated.

A more complete and complex reconstruction of the number of Jewish Holocaust survivors in the Netherlands, by age and sex, is given by Van Imhoff et al. (2001). They used two estimation procedures, a forward projection 1941–1945 and a backward projection 1966–1945. The starting point in the forward projection is a statistical overview of Jews registered in 1941 (van de Bevolkingsregisters 1942). Added to this overview is information from several other sources about the number of Jews deported and murdered, estimations of births and ‘natural’ decline between 1941 and 1945, and estimations of returnees and migrants. In the backward projection, their starting point were the findings from a demographic study conducted in 1966 combined with the Dutch national age- and sex-specific mortality rates of the 1950s and early 1960s to construct the enumerated Halachically (according to Jewish law) Jewish population in 1945. This backward reconstruction is used as a second estimate by age and gender of the number of survivors living in the Netherlands in 1945. The presented population pyramid of Jewish Holocaust survivors by Van Imhoff et al. (2001) showed persons aged 25–45, and those aged 5–7 are overrepresented; it shows hardly any gender difference.

Houwink ten Cate (1989) aimed to construct a demographic profile of survivors by investigating about 700 Jews caught in hiding in Amsterdam in 1942 and 1943. His underlying assumption was that if some demographic characteristics were overrepresented among arrested Jews in hiding, these characteristics are also likely to be overrepresented among Jews who survived in hiding. Based on frequency of characteristics of arrested Jews, gender was not unbalanced, and non-Dutch and wealthy Jews were not overrepresented. Only the 21–40 age group was overrepresented. Houwink ten Cate (1989) thus concluded that survival chances differed according to age.

For the Dutch city of Groningen and the Dutch provinces of Utrecht and Limburg, individual-based studies show some socio-demographic differences in Holocaust survival. A multivariable logistic regression on 3900 Jews living in Utrecht showed that being a woman, being young or being in the highest social class increased chances of survival (Croes 2001). A similar analysis on 2500 Jews living in Groningen (Croes and Tammes 2006: 43–63) showed that intermarried Jews and Jews in the two highest social classes had higher survival chances. Van Rens (2013: 354–355) presented *t* test statistics on socio-demographic differences in survival among 1400 Jews living in Limburg. For age, especially Jewish children aged 0–10 showed the highest proportion of survivors, and for nationality Polish Jews showed the highest proportion of survivors and German Jews the lowest.

In their study on differences in local survival rates, in a multilevel analysis, Croes and Tammes (2006) used data from about 100 retrieved original municipal lists of Jewish inhabitants. While their focus lay on associating local characteristics with local variation in Jewish survival rates, they included two individual socio-demographic characteristics: age and nationality. They found that being older increased the chances of survival though this increase was nonlinear, and in bigger cities German and other non-Dutch Jews had higher survival chances than Dutch Jews. In a follow-up study on Jewish immigrants in the Netherlands during the Nazi occupation using multivariable logistic analyses, Tammes (2007a) found that Dutch native Jews had lower survival chances than immigrants, especially among men and children, and those in Amsterdam. In another study focusing on the importance of social capital using a sample of Jews living in Amsterdam, Tammes (2007b) showed the importance of non-Jewish connections for surviving the Holocaust.

Although these findings might give us an indication of socio-demographic differences in survival, most of them are based on incomplete, aggregated, estimated, limited, local/regional data or a sample. These findings might thus not represent socio-demographic differences in survival in Amsterdam or in the Netherlands. In the next section, therefore, key hypotheses are formulated on socio-demographic differences in survival that will be tested later on in this study using individual-level data on Amsterdam Jews.

### Hypotheses

In this section key hypotheses are formulated on survival chances for different socio-demographic groups based on their opportunities and motives. A Jew’s living condition or personal characteristics and Nazi policies could have created opportunities to escape and survive persecution. Deported men were more likely to be selected by the Nazis to work in concentration and extermination camps than women (e.g. Presser 1965, part 2: 414, 426). The *gender hypothesis* is that men had higher survival chances than women. Finding a hiding place for Jewish babies or children under age 6 might have been easier because they did not need to wear a yellow Star of David. Babies could also be hidden in something portable and carried unnoticed (Flim 2005: 51, 60). Although Jewish children aged 6–14 had to wear the yellow Star of David introduced in May 1942, they did not need to have the identity card introduced in July 1941 for all Dutch citizen 15 or older and marked with a big black ‘J’ for Jews in January 1942 (Herzberg 1978: 49); this might have provided them with a better opportunity to go into hiding. The *age hypothesis* is that children aged 0–14 had higher survival chances than older Jews.

Before the Nazis occupied the Netherlands, many German Jews worked at the Dutch Committee for Jewish refugees in the 1930s. During the occupation this Committee, which became part of the Jewish Council, had the power to exempt Jews temporarily from deportation to concentration and extermination camps. German Jews and their relatives received more of these exemptions (*Sperre*) (Moore 1997: 216–219), possibly allowing them more time to find a place to hide or flee. German Jews also held advantageous positions in Westerbork to avoid or postpone deportation for themselves and their relatives (Mechanicus 1989). The *nationality hypothesis* predicts that especially German Jews had higher survival chances.

Non-Jewish relations were essential to hide and escape persecution (De Jong part 6: 45, 50). Jews who had left Judaism might have had more non-Jewish connections. The *religion hypothesis* is that Jews who had abandoned Judaism had higher survival chances than Jews who belonged to an Israelite congregation. In September 1942 intermarried Jews had to register again to get exemption from deportation, although intermarried men without children were excluded. Intermarried Jews who had not re-registered ran the risk of being deported; their Gentile partners would then have to act promptly and fill out forms mentioning that their Jewish partner had been sent to Westerbork (Tammes 2009). These non-Jewish family members could also provide other support, such as hiding places, to avoid deportation. The *mixed*-*marriage hypothesis* is that intermarried Jews had higher survival chances.

Social class might have impacted survival chances due to income and networks. Persons in higher social classes generally have more financial resources and a more diverse network. These financial and social resources might have resulted in better escape opportunities. The *social class hypothesis* is that the highest social classes had better survival chances.

The number of Jewish immigrants in the Netherlands grew especially after 1933 (van de Bevolkingsregisters 1942), and with it the proportion of non-Dutch-born Jews. Though Jewish immigrants might have had fewer social and material resources than their Dutch-born counterparts, those immigrants could have been more aware of the Nazis’ intentions and more eager to hide or flee. The *immigrant hypothesis* is that immigrants had higher survival chances than Dutch-born Jews. For families it was harder to find a hiding place without splitting up, and some might have preferred being transported to Westerbork as a complete family rather than hide separately (Presser 1965, part 2: 260). The *family hypothesis* is that married adult Jews and unmarried non-adult Jews had lower survival chances than widowed, divorced and unmarried adult Jews.

## Sources and Matching Procedure

### Nazi Registration of Jews in 1941: Retrieved Amsterdam List of Jews

In January 1941, the Nazis ordered all persons living in the Netherlands who had one or more Jewish grandparents to be registered. Amsterdam residents had to pick up their registration form in alphabetical order by last name between 10 and 18 February at special appointed locations, and return the completed questionnaire between 10 March and 7 April (Stuldreher 2007: 80–81); questions were included about the personal religious denomination of individuals, their spouses and their grandparents. The completed returned questionnaires were compared to the information in the Amsterdam population registry, and the registry cards of Jewish persons were marked with a ‘J’ and signalled with a clip (Stuldreher 2007: 79; Tammes 2009). An overview of the van de Bevolkingsregisters (1942) dated October 1, 1941 mentions 86,291 Amsterdam residents having one or more Jewish grandparents, among them 79,497 persons classified as ‘full Jews’ (e.g. Presser 1965, part 1: 62).^1^ Those ‘full Jews’ were persecuted by the Nazis; in this article they are referred to as Jews.

Dutch historians generally believe that practically all Jews complied with the order to register (De Jong 1969–1991 part 4: 874–875; Herzberg 1978: 50; Presser 1965, part 1: 62–63). Most Jews were known in their social network as being Jewish or of Jewish origin. At that time Jews could hardly have anticipated the life-threatening measures that would be taken by the Nazis in the years to come and therefore had little reason to refuse registration, particularly as citizens’ religious denomination was commonly recorded in municipal registries and reported at each decennial census.

After the liberation the marked Jewish registry cards were replaced by unmarked cards (Croes and Tammes 2006: 34), and in the post-war corrected registry one cannot trace assimilated Jews, i.e. persons who had three or four Jewish grandparents but had abandoned Judaism. Croes and Tammes (2006) recovered an undated registration list of Amsterdam Jews that mentions a total of 77,238 persons.^2^ This list contains the name, date and place of birth, marital status, address, religious affiliation, nationality and occupation of each Jew. As the youngest person on the list was born on May 7, it might be assumed that this list was being finished in the second week of May 1941 (e.g. Tammes 2011). Although this retrieved list is missing 2259 Jews when compared to the statistical overview of the Van de Bevolkingsregisters (1942), it is the only source on the Dutch capital containing information on tens of thousands of individual Jews. The question is whether these 2259 persons are a specific group that might be underrepresented on the retrieved registration list.

Among those missing persons could be the group of about 400 young adult men who were caught in a roundup on February 22, 1941 as a response to Jewish resistance, in a harassment by the uniformed commando group of the Dutch National-Socialist Party (NSB) and German patrol groups that resulted in the death of a NSB member. All 400 men were deported to Buchenwald at the end of February, and those who were still alive in May 1941 were transferred to Mauthausen (Presser 1965, part 1: 86–88). These Jews, however, are registered on the retrieved Amsterdam list as we will see later on when deceased are split up by place of death and year of death. They probably had already picked up their registration form the week before they were caught, and this form might have been returned by a household or family member.

To identify possible missing or underrepresented groups, the retrieved Amsterdam list^3^ is compared with the overview of the van de Bevolkingsregisters (1942) and a statistical overview of Jews in Amsterdam given by Veffer (1942) on the following four characteristics: gender, age, nationality and religious affiliation. According to the van de Bevolkingsregisters (1942: 22–23), 52.2 % of Amsterdam Jews were females. Based on the maiden name of married women and the first name, 51.5 % of the Jews on the retrieved list were women. Females are slightly underrepresented on the retrieved Amsterdam list. The age distribution based on the retrieved Amsterdam list hardly differs from the overview given by Veffer (1942); the biggest difference is 0.3 % in the 50–59 age group for females and 0.4 % in the 10–19 age group for males. Based on the nationality given on the retrieved Amsterdam list, 86.5 % had the Dutch nationality—very close to the 86.8 % Veffer (1942) calculated. The list also counts 8.7 % German, 2.7 % stateless, 1.3 % Polish and 0.7 % other Jewish nationals. Those percentages are again very close to Veffer’s calculations.

Although all persons on the retrieved Amsterdam list had three or four Jewish grandparents, they themselves might have not longer belonged to an Israelite congregation. Based on the religious denomination given on the retrieved Amsterdam list, 91.8 % belonged to an Israelite congregation, the same percentage given by van de Bevolkingsregisters (1942: 22–23). The retrieved list counts 7.4 % religiously unaffiliated persons and 0.7 % belonging to another congregation, nearly all converted to Christianity, as confirmed by the van de Bevolkingsregisters (1942). Although the retrieved Amsterdam list is missing 2259 Jews compared to the overviews of the van de Bevolkingsregisters (1942), it is not biased with respect to gender, age, nationality or religious denomination. This retrieved Amsterdam list is therefore an excellent source for answering the research questions.

### Lists of Victims of the Holocaust

A listing of all Jews who were deported from the Netherlands and perished without a grave is published in the book *In memoriam*-*Lezecher* (*IM*) (1995) as a means of honouring the memory of those who did not have a proper burial. This memoir contains the names, date and place of birth, and date and place of death of over 101,000 Jews. These data were gathered by the Red Cross, the Dutch Institute for War Documentation, and the Dutch Ministry of Defence and Ministry of Foreign Affairs, and checked against the population registries. The adjustments published in two addenda in 1997 and in 2000 are included in the digitised version of *IM* used in this study.

Not all Jews who perished during World War II are mentioned in *IM* though. Jews who died in the Dutch concentration and transit camps Westerbork and Vught, and those who perished outside a Nazi camp or who had a grave are not mentioned in *IM*. For this reason, lists of Jews who died in Westerbork or Vught and buried Jews mentioned in other death lists are put in a different victimisation database (WB+), counting in total more than 1200 Jews.^4^

I also had access to a database containing data extracted from the website Digital Monument to the Jewish Community in the Netherlands (DMJ).^5^ DMJ is an Internet monument dedicated to preserving the memory of all those who were persecuted as Jews during the Nazi occupation of the Netherlands and did not survive. Since its launch in 2005 this website is continuously updated. The DMJ database contains information such as first, last and maiden name, date of birth, and last official place of residence on more than 104,000 Jews, including those who died in Westerbork and Vught and other Dutch locations, as well as recent corrections to *IM*.

The victims mentioned in those databases overlap significantly. However, as the listings’ initial purposes to gather and publish information on victimisation of the Holocaust differ, the databases might also differ slightly in terms of the victims listed, and none of the lists will be complete. To minimise underreporting of specific groups of victims and to avoid ‘political’ biases (Aronson et al. 2013: 290; Brunborg et al. 2003: 236–237), this study uses all three databases to determine who among Amsterdam Jews fell victim to the Holocaust.

### Lists of Survivors

Amsterdam Jews not matched to the lists of victims or reported as missing on the victim lists were further investigated by matching them to other post-war information (e.g. Brunborg et al. 2003). Ideally, these Jews would be checked against the names on the Dutch post-war population registry to determine whether they were alive after the liberation. However, this registry is not computerised and is not easy accessible due to privacy legislation. In addition, not all survivors returned to their place of residence or to the Netherlands. Instead of the registry, this study uses a database of Jewish Holocaust survivors who had lived in the Netherlands during the Nazi occupation. Lists of returnees and camp survivors had been put in a database, counting 22,692 Jewish survivors.^6^ Some survivors were registered on multiple lists. After identifying multiple registered persons using surname/initials, given name and/or date of birth, the database counted 16,704 unique persons. In addition to surname, last name and date of birth, this database contains information on place of birth and former or last place of residence, although it is unclear what that means exactly. However, for many persons, data are missing. For about half no former or last place of residence is given, and for more than half no place of birth is mentioned.

The 16,704 persons in the survivors database are far lower than the estimated number of survivors since, among others, the returnee registry is far from complete (Van de Vosse 1947). Hence, this database can only account for part of the Jews not matched to victimisation lists who had shown signs of life after the liberation. Matching might also be useful to determine which groups of Jews are underrepresented in the Dutch survivors database according to their socio-demographic profile.

### Matching Amsterdam Jews to Lists of Victims

To compare the Jews mentioned on the retrieved Amsterdam list to Jews mentioned in the victimisation databases, this study uses a deterministic linkage approach by constructing a unique matching key (e.g. Grannis et al. 2002). To this end, only individual characteristics present in both databases can be used. Although Amsterdam Jews were asked to give their complete first and last name when registering in 1941, it might be better not to use the complete name to avoid mismatching due to possible different spelling or typos. Following up on the matching method developed by Croes and Tammes (2006), this study uses the first two characters of the first name and the first two characters of the last and maiden name of Jews registered on the Amsterdam list that were entered into a database, but excludes the prefix of the surname, like *van* (‘of/from’), *de*/*het*/*’t* (‘the’), *der* (‘of the’), as such prefixes are quite common. The combination of these two components is not that unique and produces many double matches; to avoid these, the complete date of birth is added. Married women also undergo a matching that includes the first two characters of the maiden name instead of the last name.

This developed matching key appeared to be very unique: only 76 combinations exist twice. This means that 152 Jews (0.2 % of all Amsterdam Jews) could not be uniquely identified on the Amsterdam list. These 152 persons were manually checked to see if they had perished during the Nazi occupation using the described victimisation databases. For 210 Jews it was impossible to construct this matching key due to a missing value on one or several key components. Moreover, 112 persons appear twice on the Amsterdam list. In total, 76,916 (77,238-210-112) individual records were matched using the constructed matching key. The matching procedure is repeated three times, first matching Amsterdam Jews to *IM*, then to WB+ and finally to those mentioned in DMJ who resided in Amsterdam.

The non-matched Jews were subjected to a second matching procedure using an alternative matching key. Sometimes the date of birth was not too readable on the Amsterdam list, so an alternative matching key was constructed to perform a second matching procedure: first two characters of the first name, first two characters of the last name, and a combination of two of the three date-of-birth components: day, month and year. This resulted in three alternative matching keys for each person, namely the first two characters of the first name, the first two characters of the last name, and, respectively, day and month, day and year, or month and year. The results of this second matching procedure were checked manually.

Using a deterministic linkage approach, these matching procedures resulted in 58,144 matched individual records; 95.7 % were mentioned in both *IM* and DMJ (Table 1). Another 3.5 % were from DMJ only. All the matched persons found in WB+ were also found in DMJ or *IM*; only a very small number were found in *IM* only. Among the matched individuals 58,070 were matched to DMJ and all of them lived in Amsterdam.

**Table 1:** Results of matching procedures

Sources	Total	
	*N*	%
IM	72	0.1
DMJ	2007	3.5
WB+	0	0
IM & DMJ	55,650	95.7
IM & WB+	2	0.0
DMJ & WB+	246	0.4
IM & DMJ & WB+	167	0.3
Total	58,144	100.0

*IM* In memoriam-Lezecher

*DMJ* Digital Monument to the Jewish Community in the Netherlands

*WB+* Lists of Jews who died in Westerbork or Vught and buried Jews mentioned on other death lists

### Matching Amsterdam Jews to Lists of Survivors

To compare Amsterdam Jews who were not matched to lists of victim to survivor lists, the same matching key is used as described before. Within the Dutch survivors database, 2332 of the 16,704 Jews had an incomplete key due to missing information and 410 were born after the estimated registration date of the Amsterdam list (May 1941), resulting in 13,962 Jewish survivors to be included in the matching procedure. None of the Jews reported missing were matched to the survivor database. Using the matching key, 2878 of the 18,772 Jews not matched to victim lists were matched to the survivor lists (15.3 %).

For more than half of the 2878 matched persons, no last place of residence was given and for about one-third (about one thousand) Amsterdam was their last place of residence. Since about 2600 survivors had Amsterdam as last place of residence, using the alternative matching key described earlier the Amsterdam Jews not matched to victim lists once again are matched to the remaining 1600 survivors who had lived in Amsterdam. This yielded another 216 matched individual records resulting in 3094 matched survivors, which is 16.5 %. This low percentage of matched records might be due to the incompleteness and inaccuracy of the information on the survivor lists.

Cross-tabulations and Chi-square test show that for nationality German and Polish Jews were overrepresented and Dutch Jews underrepresented, and for civil status unmarried Jews were overrepresented and divorced, widowed and married Jews were underrepresented among the Jews matched to the survivor lists. Among married Jews, intermarried Jews were under-registered. The elderly and females were underrepresented, while no difference in post-war registration exists according to social class.

## Survival Rate Among Amsterdam Jews

**Figure 1:**
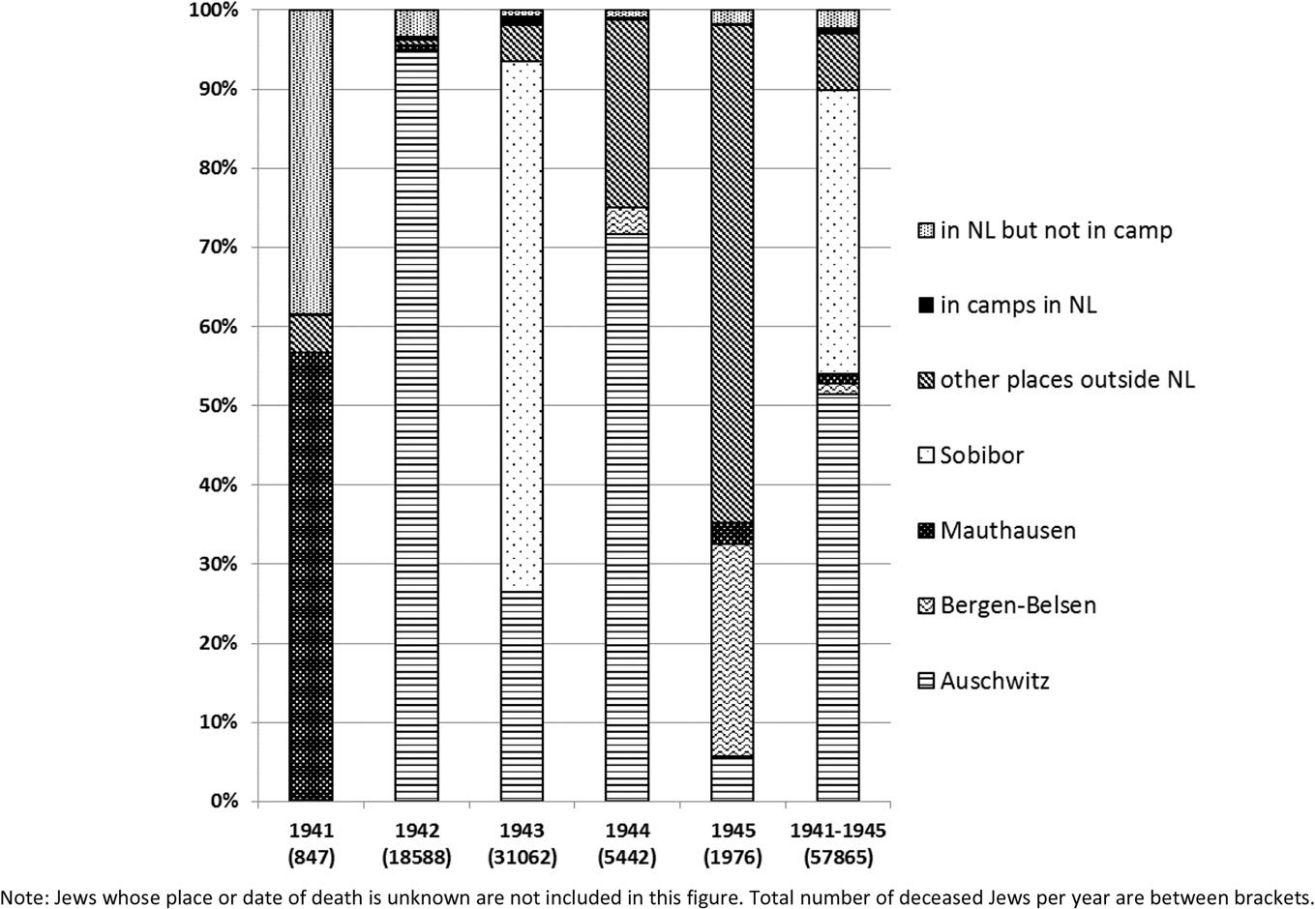
Percentage of deceased by place of death and year of death

Victims of the Holocaust are Jews who were directly or indirectly killed by the Nazis during the World War II. Following Tabeau and Bijak (2005) and Van Imhoff et al. (2001), it is useful to distinguish several death categories according to place and date of death, and if possible causes of death. This study therefore makes a distinction between those who died abroad, after being deported to concentration and extermination camps or while fleeing to safer countries, and those who died in the Netherlands. About half of the deceased Amsterdam Jews perished in Auschwitz and about one-third in Sobibor (Fig. 1, last bar). The Sobibor victims died all in 1943 (Fig. 1, third bar). Although only a small percentage of Amsterdam Jews died in the Netherlands (Fig. 1, last bar), this latter category is split into two subcategories to make a more precise estimate of Jewish victimisation: those who died in Dutch locations that had a transit or concentration camp such as Westerbork, Vught, Barneveld and Amersfoort (Leusden), and those who died in other Dutch locations.

Van Imhoff et al. (2001) estimated the number of Jews in the Netherlands who died of natural causes between October 1941 and May 1945 at around 2000. Those who died in Dutch locations other than Westerbork, Vught, Barneveld and Amersfoort (Leusden) might have died due to natural causes such as old age or illness, some while in hiding (Presser 1965, part 1: 274–276). Others committed suicide (e.g. Presser 1965, part 1: 284–186). Ultee and Luijkx (1997) counted 251 Amsterdam Jewish residents who committed suicide between 1941 and 1943. Using information on cause of death obtained from the DMJ website (e.g. Braun 2016), this study determined that 268 Amsterdam Jewish residents had committed suicide between 1941 and 1945.^7^ Jews who committed suicide and those who died in Westerbork, Vught, Barneveld and Amersfoort (Leusden) will be counted as victims of the Nazi occupation. Furthermore, 46 Jews were killed by the Nazis in the Netherlands when trying to escape deportation or after being arrested as a resistance member.

The number of Amsterdam Jews who were victims of Nazi persecution within the Netherlands is 685 (Table 2, rows C, D, E), and outside the Netherlands 56,188 (Table 2, row A). This is excluding 180 Jews whose place of death is unknown but who may have died abroad, and 80 missing persons of whom we might assume 70 years after the end of World War II to have been killed abroad (Table 2, rows B, H). If we include those with an unknown place of death and those reported missing, adding the number of Jews who were victims of Nazi persecution within and outside the Netherlands (Table 2, rows A, B, C, D, E, H) yields a total of 57,133 Jewish victims.

**Table 2:** The fate of Jews registered in Amsterdam in May 1941

	Victimisation	Numbers	%
A	Died abroad (mostly in camps after being deported)	56,188	73.1
B	Place of death unknown	180	0.2
C	Died in camps in NL	371	0.5
D	Committed suicide in NL	268	0.3
E	Killed by Nazi in NL but outside camps	46	0.1
F	Assumed to have died of natural causes May 1941–June 1942	578	0.8
G	Assumed to have died of natural causes July 1942–May 1945	433	0.6
H	Missing	80	0.1
I	Not reported as having died during WWII	18,772	24.4
J	Total	76,916	100.00

When counting all the Jews on the retrieved Amsterdam registration (77,238), except for the 210 persons for whom we miss a part of their name or date of birth and the 112 double-listed persons, the victimisation rate is 74.3 % (57,133/76,916). Excluding from the denominator those who died of natural causes in the Netherlands before the start of the regular deportations in July 1942 (Table 2, row F) results in a victimisation rate of 74.8 % (57,133/(76,916-578)); excluding those who are assumed to have died of natural causes after June 1942 (Table 2, row G) results in a victimisation rate of 75.3 % (57,133/(76,916-578-433)). Depending on the size of the denominator—who to include that ran the risk of becoming a victim of Nazi persecution—the Amsterdam Jewish victimisation rate lies between 74.3 and 75.3 %. This means that between 24.7 and 25.7 % of Amsterdam’s Jews survived the Holocaust, though only for a small proportion this study did find evidence that they were alive at the end of the war due to incompleteness and inaccuracy of the survivors database. The Amsterdam survival rate is lower than the Dutch national average of 27.1 % (Hirschfeld 1991), as many other Dutch cities show more favourable survival rates (Croes and Tammes 2006: 39–41).

## Measuring Socio-demographic Differences in Survival

As socio-demographic characteristics may be interrelated, age, gender, nationality, immigrant status, religious affiliation, civil status and social class were included in multivariable analyses to investigate their joint effect. First a logistic regression model is used to measure the strength of the effect of socio-demographic characteristics on the chances of survival, expressed in odds ratios with a 95 % confidence interval. The dependent variable in this analysis is whether someone perished during the Holocaust or survived the Holocaust. Due to some missing values on one or several variables for 393 Jews, a total of 75,512 Jews were included in this analysis. P-values and standard errors were adjusted for family relationships by taking household as a cluster, taking into account the covariance between members within a household. Using the intercept and the regression coefficients, adjusted survival chances are calculated.

Second, a Cox regression model (Cox 1972) is used to further investigate whether the socio-demographic factors described were associated with higher or lower risk of death due to the Holocaust. The Cox model offers increased power of analysis by including information on date of death. Unlike the logistic regression, it gives greater weight to deaths that occurred earlier in the period versus deaths that occurred later. The observation period for all Jews in the analysis starts on February 1941; that month about 400 Jewish men were caught during a raid and deported to concentration and labour camps Buchenwald and Mauthausen, and some of them already perished in the next month (see Fig. 1). The observation period ends when someone died because of Nazi persecution, or was censored because of natural death before May 1945 when Germany surrendered and the Netherlands was liberated. For most of the Jews, the month and year of death are known. However, for 247, the month of death is unknown and they are excluded from the analysis. Moreover, 32 Jews died between June and December 1945. Although they died after the German surrender, it is assumed that their death is directly related to their suffering during World War II. Hence, they are counted as Holocaust victims and registered their date of death as May 1945.

Table 3 shows the descriptive statistics for both analyses. There were slightly more females than males, about 13 % had a non-Dutch—mostly German—nationality, and about 8 % had abandoned Judaism. A large majority, about 86 %, belonged to the Dutch Israelite congregation, and about 5 % belonged to the Portuguese Israelite congregation. About 5 % were younger than age 6 and about 32 % were aged 31–50 in May 1941. Using the civil status on the registration list, about 27 % of adult Amsterdam Jews (aged 18 and over) were either widowed, divorced or unmarried; the other 73 % were married adults or unmarried non-adults (under-18 s). As non-Jews were not included in the list, married Jews living at an address where only one married person was listed are coded as having a non-Jewish spouse, although some Jewish spouses may have been temporarily living elsewhere. Over 6000 Jews, or about 8 %, were identified as intermarried −15.8 % of all married Jews. This percentage is close to the percentage of intermarried Jews in the 1930s based on other administrative sources (e.g. Tammes 2010).

**Table 3:** Descriptive statistics of the population

Variable	Logistic regression		Cox regression	
	*N*	Pct.	*N*	Pct.
*Survival*				
Perished	56,928	75.4	56,681	74.3
Survived	18,584	24.6	18,584	24.4
Right censored (died in NL of natural causes)			1003	1.3
*Gender*				
Male	36,600	48.5	37,004	48.5
Female	38,912	51.5	39,264	51.5
*Age*				
0–5	4145	5.5	4147	5.4
6–14	7405	9.8	7399	9.7
15–30	18,289	24.2	18,257	23.9
31–50	24,148	32.0	24,175	31.7
50+	21,523	28.5	22,290	29.2
*Immigrant*				
Born in the Netherlands	64,397	85.3	65,138	85.4
Born abroad	11,115	14.7	11,130	14.6
*Nationality*				
Dutch	65,398	86.6	66,184	86.8
German	6589	8.7	6585	8.6
Stateless	2049	2.7	2027	2.7
Polish	974	1.3	971	1.3
Other	502	0.7	501	0.7
*Religion*				
Portuguese Israelite congregation	4080	5.4	4124	5.4
Dutch Israelite congregation	65,297	86.5	65,997	86.5
Secular Jews	5627	7.4	5641	7.4
Converted Jews	508	0.7	507	0.7
*Family stage*				
Married adults and unmarried non-adults (18−)	55,013	72.9	55,568	72.9
Divorced, widowed and unmarried adults (18+)	20,499	27.1	20,700	27.1
*Mixed marriages*				
Married to a non-Jew	6176	8.2	6279	8.2
*Social class*				
Higher managers and professionals	1395	1.8	1407	1.8
Lower managers, professionals, clerical and sales	15,854	21.0	15,953	20.9
Foremen and skilled workers	5643	7.5	5689	7.5
Farm workers, farmers and fisherman	61	0.1	62	0.1
Lower-skilled workers	8149	10.8	8160	10.7
Unskilled workers	3598	4.8	3600	4.7
Unclassified	147	0.2	149	0.2
No job	40,665	54.0	41,248	54.1
Total	75,512	100.0	76,268	100.0

Occupations were coded in accordance with the Historical International Standard Classification of Occupations (HISCO) scores and separated into social classes (Van Leeuwen et al. 2004). The main dimensions of social class encompass the distinction between manual and non-manual labour, as well as skill level, supervisory responsibility and economic sector. Originally, HISCO comprised twelve HISCLASSES (Van Leeuwen and Maas 2011). To avoid small numbers in some classes, this study adopted the condensed version of seven HISCLASSES. Next, all farmers and fishermen were put into one group (only a small group had an agrarian occupation), and all without a job (including children) were coded jobless. Among Amsterdam Jews <2 % had a job in the highest social class (higher managers and professionals). About 21 % of the working Jews had a job in the second highest class. About 7 % were skilled workers, nearly 11 % lower-skilled workers, and nearly 5 % unskilled workers. More than half of Amsterdam Jews were jobless.

### Results of Logistic Regression

The intercept in Table 4 shows a survival chance of 24.4 % for the reference group: jobless married women/unmarried girls aged between 15 and 30, born in the Netherlands who were Dutch nationals and belonged to the Dutch Israelite congregation. The effect of male Jews was not significant, indicating that males did not have better or worse survival chances than females. This rejects the gender hypothesis. The impact of age on surviving the Holocaust was significant. The youngest age group had significantly better survival chances than the reference category (aged 15–30), but the other age groups, including ages 6–14, had significantly lower survival chances, which partly rejects the age hypothesis. If the reference group were Jews aged 0–5 instead of 15–30, the survival chances would be 28.3 %.

**Table 4:** Estimates of odds ratios from logistic regression model for the association between socio-demographic characteristics and survival of the Holocaust

	OR	95 % CI	*P* value factors	*P* value parameters	Survival chances
Intercept	0.32	0.31, 0.34	<0.001		24.4
*Gender (ref.* = *female)*					
Male	1.01	0.97, 1.05	0.735	0.735	24.3
*Age (ref.* = *15*–*30)*					
0–5	1.23	1.13, 1.34	<0.001	<0.001	28.3
6–14	0.75	0.70, 0.82		<0.001	19.6
31–50	0.64	0.61, 0.68		<0.001	17.2
50+	0.36	0.34, 0.39		<0.001	10.5
*Immigrant (ref.* = *born in NL)*					
Born abroad	1.59	1.45, 1.74	<0.001	<0.001	33.8
*Nationality (ref.* = *Dutch)*					
German	1.18	1.05, 1.33	<0.001	0.004	27.6
Stateless	1.29	1.11, 1.51		0.001	29.4
Polish	1.51	1.22, 1.86		<0.001	32.7
Other	3.05	2.32, 4.03		<0.001	49.7
*Religion (ref.* = *Dutch Israelite cong.)*					
Secular Jews	2.95	2.74, 3.18	<0.001	<0.001	48.9
Converted Jews	6.06	4.67, 7.86		<0.001	66.2
Portuguese Israelite congregation	1.06	0.95, 1.18		0.277	25.5
*Mixed marriages*					
Married to non-Jew	5.34	5.00, 5.70	<0.001	<0.001	63.3
*Family (ref.* = *married and children)*					
Divorced, widowed and unmarried adults (18 +)	1.44	1.37, 1.51	<0.001	<0.001	31.7
*Social class (ref.* = *no job)*					
Higher managers and professionals	1.81	1.59, 2.06	<0.001	<0.001	36.9
Lower manager, professional, clerical and sales	1.12	1.07, 1.19		<0.001	26.7
Foremen and skilled workers	0.55	0.51, 0.60		<0.001	15.2
Farm workers, farmers and fisherman	0.62	0.30, 1.26		0.188	16.7
Lower-skilled workers	0.56	0.52, 0.60		<0.001	15.2
Unskilled workers	0.46	0.42, 0.51		<0.001	13.0
Unclassified	3.04	2.11, 4.39		<0.001	49.6

*Ref*. reference group, *OR* odds ratio, *CI* confidence interval. Adjusted for family relationship by taking into account clustering for household

The impact of being an immigrant on surviving the Holocaust was significant. Jews born abroad had significantly better survival chances than Dutch-born Jews, which supports the immigrant hypothesis. The impact of nationality on surviving the Holocaust was significant. All Jews with a non-Dutch nationality had better survival chances than Dutch-Jewish nationals, but German or stateless—mainly former German—Jews did not have the highest chances, which partly rejects the nationality hypothesis.

The impact of religious denomination on surviving the Holocaust was significant. Secular and converted Jews had significantly better survival chances than Jews belonging to the Dutch Israelite congregation, which supports the religion hypothesis. Intermarried Jews also had significantly higher survival chances, which supports the mixed-marriage hypothesis.

The impact of family stage on surviving the Holocaust was significant. Married adults and unmarried non-adults had significantly lower survival chances than divorced, widowed and unmarried adults, which supports the family hypothesis.

The impact of social class on surviving the Holocaust was significant. Jews in the two highest social classes had significantly better survival chances than jobless Jews, while the other social classes, except for the small group of farmers and fishermen, had significantly lower chances. This result supports the social class hypothesis.

Since Jewish migrants from both Antwerp and Paris were overrepresented among the deported Jews and had lower survival chances than native Jews, it might be worthwhile to elaborate on the survival chances of Jewish immigrants living in Amsterdam by adding interactions to the model (see supplementary Table 1 in the appendix for full details). The interaction between immigrant and gender is not significant. The interaction between immigrant and age shows that the advantage of immigrant Jews is especially marked in the 6–14 age group. Though the highest social class was protective for both immigrant and native Jews, the interaction between immigrant and social class shows that the highest social class was less protective for immigrant Jews. By contrast, the social classes of lower-skilled workers and unskilled workers were more protective for immigrant Jews.

As especially adult men were favoured during selection for work in concentration and extermination camps that might increase their chances of surviving the Holocaust, an interaction between gender and age is included (see supplementary Table 1, model 2). While men aged 15–30 had lower survival chances than women in that age group, the disadvantage of being male in the 15–30 age group is not so marked among the other age groups.

### Results of Cox Regression

**Figure 2:**
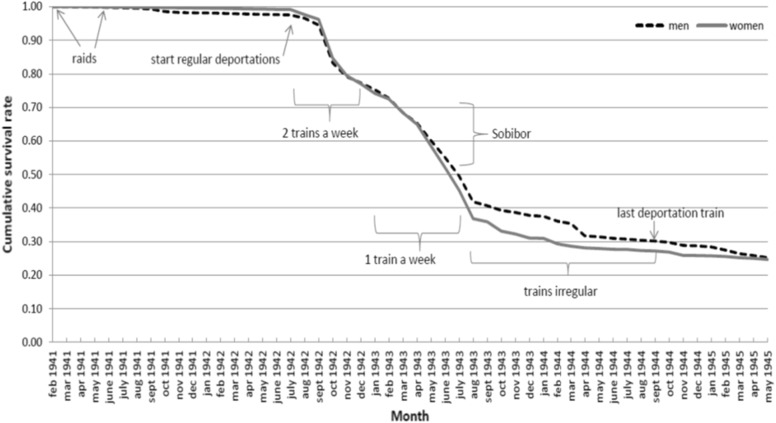
Survival functions of male and female Amsterdam Jews

Figure 2 shows the survival functions for male and female Amsterdam Jews between February 1941 and May 1945. The first of a total of 103 deportation trains left transit camp Westerbork on 15 July 1942. In the second half of 1942 there were about two weekly trains to Auschwitz. From early 1943 onwards about one train a week left Westerbork, between March and June 1943 all trains went to extermination camp Sobibor. After the summer of 1943 trains went irregularly and also to other destinations such as Bergen-Belsen and Theresienstadt; the last deportation train left the Netherlands in September 1944 (Hirschfeld 1991). The slope of the survival function shows varying steepness over time, affecting survival rates accordingly. The varying survival chances over time expressed in the survival function are due to several factors, including the variation in monthly transportation of Jews from Amsterdam to Westerbork (Meershoek 1999: 247, 288), weekly number of deportation trains and number of deportees, and destination of the trains.

Since date of deportation is not given in the used sources, it cannot be included in the Cox regression model. Due to missing values for some socio-demographic characteristics, the total number of Jews included in this analysis is 76,268. The Cox proportional hazard assumption was tested using the survival plots for each variable in turn. A time-varying component was added for age which demonstrated changes in relative risk between age categories over the period of study. The results of the Cox regression are given in Table 5. The risk of death among male Jews was reduced by 3 % compared with female Jews. Results from the logistic regression analysis showed no significant difference in survival chances between males and females, based on who was alive in May 1945 (Table 4). Cox regression, however, takes into account the stronger decrease in survival function among female Jews in 1943 and 1944 (see Fig. 2), resulting in a significant higher hazard rate for females. Jews aged 0–5 showed a higher hazard rate than Jews aged 15–30, but the higher risk for this age group decreased over the course of the Nazi occupation, adding information to the results from the logistic regression analysis. Jews aged 6–14 showed a lower hazard rate than Jews aged 15–30, but the risk for this age group increased over the course of the Nazi occupation. The risk among the oldest age group did not differ from Jews aged 15–30 though their risk increased over the course of the Nazi occupation; this result differs from the logistic regression analysis as it showed worse survival chances for the oldest age group. The risk of death among immigrants was reduced by 25 % compared with Dutch-born Jews. Non-Dutch Jews showed lower hazard rates than Dutch Jews, but German or stateless Jews did not have the lowest hazard rate. Secular Jews and converts to Christianity showed lower hazard rates than Jews belonging to the Dutch Israelite congregation. Also Jews belonging to the Portuguese Israelite congregation showed lower hazard rates than Jews belonging to the Dutch Israelite congregation; this result differs from the logistic regression analysis as it showed no significant difference in survival chances. Divorced, widowed and unmarried adult Jews showed lower hazard rates than married adults and unmarried children. The risk of death among intermarried Jews was reduced by about 59 % compared with non-intermarried Jews. The two highest social classes showed lower hazard rates than jobless Jews, while the other classes showed higher hazard rates. Those results do not differ much from the logistic regression analysis.

**Table 5:** Estimates of hazard ratios from Cox regression model for the association between socio-demographic characteristics and victimisation of the Holocaust

	HR	95 % CI	*P* value factors	*P* value parameters
*Gender (ref.* = *female)*				
Male	0.97	0.95, 0.98	<0.001	<0.001
*Age (ref.* = *15*–*30)*				
0–5	1.41	1.19, 1.67	<0.001	<0.001
6–14	0.84	0.73, 0.96		0.012
31–50	0.35	0.32, 0.39		<0.001
50+	0.93	0.84, 1.03		0.140
*Change to age HR per month* ^*a*^				
0–5	0.99	0.98, 0.99		<0.001
6–14	1.01	1.01, 1.02		<0.001
31–50	1.04	1.04, 1.05		<0.001
50+	1.02	1.02, 1.03		<0.001
*Immigrant (ref.* = *born in NL)*				
Born abroad	0.75	0.72, 0.78	<0.001	<0.001
*Nationality (ref.* = *Dutch)*				
German	0.86	0.82, 0.92	<0.001	<0.001
Stateless	0.79	0.74, 0.85		<0.001
Polish	0.77	0.69, 0.85		<0.001
Other	0.54	0.44, 0.66		<0.001
*Religion (ref.* = *Dutch Israelite cong.)*				
Secular Jews	0.52	0.49, 0.55	<0.001	<0.001
Converted Jews	0.28	0.22, 0.34		<0.001
Portuguese Israelite congregation	0.92	0.87, 0.96		<0.001
*Family (ref.* *=* *married and children)*				
Divorced, widowed and unmarried adults (18+)	0.91	0.89, 0.93	<0.001	<0.001
*Mixed marriages*				
Married to non-Jew	0.41	0.39, 0.43	<0.001	<0.001
*Social class (ref.* = *no job)*				
Higher managers and professionals	0.61	0.57, 0.65	<0.001	<0.001
Lower manager, professional, clerical and sales	0.88	0.86, 0.90		<0.001
Foremen and skilled workers	1.22	1.18, 1.26		<0.001
Farm workers, farmers and fisherman	1.15	0.86, 1.53		0.339
Lower-skilled workers	1.27	1.24, 1.31		<0.001
Unskilled workers	1.41	1.35, 1.47		<0.001
Unclassified	0.51	0.40, 0.65		<0.001

*Ref.* reference group, *HR* hazard ratio, *CI* confidence interval. Adjusted for family relationship by taking into account clustering for household

^a^Time varying covariate for age to satisfy proportional hazards assumption of the Cox model

## Post-war Socio-demographic Profile

**Figure 3:**
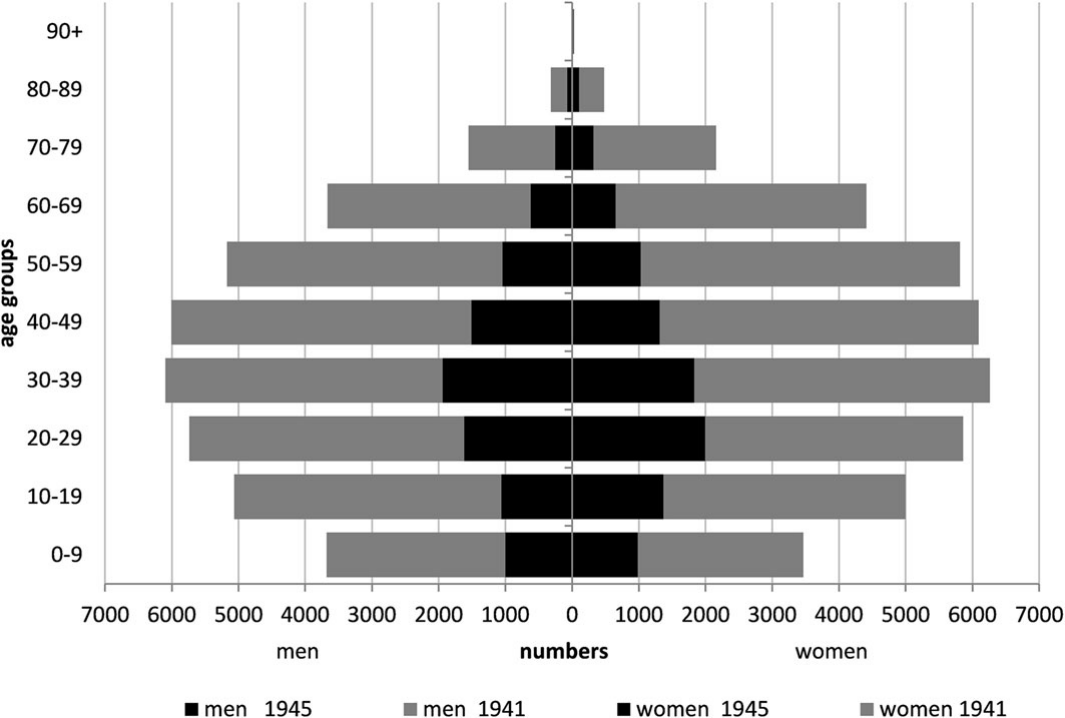
Population pyramids of Jews in Amsterdam, May 1941 and May 1945

Using the Amsterdam registration list and results from the matching procedure, it is possible to construct population pyramids of the Amsterdam Jewish population in 1941 and 1945; these pyramids do not include babies and young children born after May 1941. Even though not all survivors returned to or stayed in Amsterdam after the liberation, the population pyramid for 1945 shows the destruction of Amsterdam Jewry compared to the pyramid of the Jewish population in 1941 (Fig. 3). Although the shape of the post-war population pyramid shows the same onion shape as the population pyramid on the eve of the Holocaust, indicating fewer children and youngsters than middle-aged persons, the further decimation of the younger groups by the Nazis constituted a potentially problematic issue in terms of building up a post-war Jewish community in Amsterdam. While the post-war Jewish population increased between 1954 and 1966, the 0–14 age group fell below the average growth, leading to further ageing of the Jewish population. This is partly due to the great Holocaust losses in the 6–14 age group, resulting in inadequate additions at the base of the post-war age pyramid (Van Praag 1976: 26–27). Other aspects such as alienation, age and gender composition, and out-marriage also underlie the socio-demographic profile (DellaPergola 1996; Schmelz 1981). The better survival chances among intermarried Jews, secular Jews and converts to Christianity, as shown previously, might therefore also have impacted the post-war socio-demographic profile of Amsterdam Jews.

## Conclusion and Discussion

This study determined the victimisation rate of Amsterdam Jewish residents on the eve of the Holocaust as well as differences in their risk of death and survival chances. To calculate victimisation rates and survival chances, ideally both victims and survivors need to be mentioned on a population list, preferably one created before the start of the violence. Upon closer inspection, the retrieved registration list of about 77,000 Amsterdam Jewish residents in early spring 1941 is a very useful population source for this study. Linking this registration to several lists of Holocaust victims resulted in a victimisation rate between 74.3 and 75.3 %, depending on who to include as potentially at risk of becoming a victim of Nazi persecution. About half of the victims were killed in Auschwitz and about one-third in Sobibor. The survivors database in its current state is unsuitable to validate the non-matched Jews to victimisation lists due to incompleteness and inaccuracy. Nonetheless, the calculation based on the linkage of the retrieved Amsterdam list with different lists of Holocaust victims provides us with the most accurate victimisation rate among Amsterdam Jews.

The created individual-level database on all Amsterdam Jews listed allowed me to conduct more advanced analyses on socio-demographic differences in survival compared to the research on aggregated data including backward estimations by Van Imhoff et al. (2001) and the local-context-focused research by Croes and Tammes (2006). This study used logistic regression to calculate differences in individual survival chances according to socio-demographic characteristics and Cox regression to calculate differences in risk of death due to the Holocaust by including information on date of death. The results of both methods are complementary towards understanding differences in survival. While men showed a lower risk of death (i.e. perished at a later date) compared with women, the two hardly differed in survival chances. This result might indicate that being selected for work in concentration and extermination camps was not protective. Though Jews aged 6–14 had a lower risk of death initially, in the end they had lower survival chances than Jews aged 15–30; for Jews aged 0–5 it was the other way around. This result indicates it might be better to formulate separate hypotheses for Jews aged 0–5 and Jews aged 6–14. The better survival chances of immigrants compared with natives might indicate that immigrants were more eager to hide or flee. In addition, German Jews showed better survival chances than Dutch Jews while Polish and other Jewish nationals showed the highest survival chances. The better survival chances of Jews in the two highest social classes compared with jobless Jews might indicate the importance of material resources and connections. The better survival chances of Jews who had abandoned Judaism compared with Jews who belonged to the Dutch Israelite congregation might especially indicate the importance of non-Jewish connections. The lower survival chances of married adults and unmarried children might indicate that families struggled with decisions on hiding or finding hiding places. And yet, intermarried Jews had one of the highest survival chances. Though these Jews were exempted from deportation, they had to obey anti-Jewish measures and Nazi policies on intermarried Jews underwent changes (e.g. Stuhldreher 2007), resulting in about 37 % of them not surviving the Holocaust.

Whereas these findings are based on Amsterdam Jews, covering more than half of the Jews living in the Netherlands, the impact of some socio-demographic characteristics on the survival of Jews living in other Dutch locations might vary due to differences in composition of the Jewish community or the local context. Nonetheless, the presented findings indicate that differences in survival chances were not random but are related to socio-demographic characteristics. Since this study had information on Jews for early spring 1941 and their date of death or being alive in May 1945, adding information on in-between events for both victims and survivors could result in testing the formulated and other hypotheses in a life-course approach, as shown in a pilot study by Tammes (2012a). Using Jewish Council index cards, information such as whether someone was exempted from deportation, date of arrival in transit camp Westerbork, and date of deportation to the ‘East’ could be added.

Although a comparison in survival of Jews in Amsterdam with those in other European cities is beyond the scope of this article, a sense of how Amsterdam Jews related to Jews in other European cities might be instructive towards studying underlying causes for differences in victimisation. In contrast to Antwerp and Paris, Jewish immigrants in Amsterdam had better survival chances than native Jews. Compared to Antwerp and Paris, a relatively higher proportion of the migrants in Amsterdam were refugees from Nazi Germany. As secular Jews had better survival chances, pre-war integration or assimilation processes among Jews might be of importance. The degree of integration is hard to compare, as clear information is lacking for Antwerp and Paris. Although Wasserstein (2012) presented some qualitative or impression-based evidence, including some crude numbers on the integration of European Jews on the eve of World War II, statistical (comparative) evidence for European cities is scarce. Ultee and Luijkx (1998) gathered (statistical) comparable information for six European cities on factors like out-marriage, residential segregation and secularisation. Gathering this information together with survival rates for other European cities would create a unique opportunity to investigate the effects of integration on survival rates in an ecological study.

Victimisation of the Holocaust has not received much attention within the field of demography of conflict and violence. This study on individual survival chances sheds light on studying demographic consequences of conflict and violence. It has shown a matching method on how to link essential sources such as population lists to lists of victims and survivors, and considerations when counting victims and survivors and calculating victimisation rates. Most studies within this field focus on counting or estimating casualties and classifying victims in relation to conflict and violence. This study went a step further and calculated a victimisation rate, and by using advanced statistical techniques determined socio-demographic differences in survival and tested hypotheses on survival. While Nazi persecution decimated the Amsterdam Jewish community, differences in survival impacted the post-war population structure of the Amsterdam Jewish community. The greater losses among Jews aged 6–14 impacted community reconstruction, while higher survival rates among assimilated Jews—those who had abandoned Judaism or intermarried—and foreign-born Jews resulted in a changed composition of the Jewish community after the war.

## Electronic supplementary material

Below is the link to the electronic supplementary material.

Supplementary material 1 (DOCX 18 kb)

## Footnotes

1 A person was considered a ‘full Jew’ if he/she had at least three grandparents who belonged to an Israelite congregation, or had two Jewish grandparents and was married to a ‘full Jew’ or belonged to an Israelite congregation.

2 NIOD, Institute for War, Holocaust and Genocide Studies, archive no. 77, inv. nos. 1411–1413. This study involved additional data entry and data cleaning using scans of the Amsterdam list provided to me by Robert Braun in January 2012.

3 For 52 persons their gender could not be determined, for 162 persons their age could not be determined, and for 75 persons their religious denomination was not given or was unreadable.

4 Several sources: (1) Dutch Red Cross, Information Office, Westerbork Archive, 'jüdische Gemeinde des Kamps Westerbork, Sterberegister'; ibidem, 'Verzeichnis von verstorbenen jüdischen Lagereinsaszen aus Vught'; (2) National Archives of the Netherlands, archive no. 2.09.34.01: inv. nos. 28, 36 and 39; (3) NIOD, Institute for War, Holocaust and Genocide Studies, archive no. 182: inv.no. 36B; (4) New Israelite Weekly [*Nieuw Israëlitisch Weekblad* (NIW)] 27 (2-3-1965).

5 For more information, visit: http://www.joodsmonument.nl/?lang=en. Robert Braun provided me with this DMJ database in June 2013.

6 Lonnie Stegink, affiliated with the Jewish Historical Museum in Amsterdam, provided me in November 2011 with a database on Jewish Holocaust survivors constructed by a group of volunteers under the supervision of Peter Landé, affiliated with the US Holocaust Memorial Museum (USHMM) and Nolan Altman, coordinator for JewishGen’s Holocaust Database. Information on this ‘Dutch survivors list’, such as sources used, can be found at http://www.jewishgen.org/databases/Holocaust/0239_Dutch_survivors_lists.html. Lonnie Stegink removed from the original database 1471 Jews who had not survived the Holocaust but were accidently included in the survivor database.

7 Searching for ‘suicide’ or ‘zelfmoord’, and in given obituaries for ‘overleed plotseling’ [died suddenly], December 2015–January 2016. This study also used information from the archives of the NIOD, Institute for War, Holocaust and Genocide Studies, archive no. 182, inv.no. 2.35 ‘zelfmoordgevallen’ (suicide cases), file 203 ‘suicide reports July 1942-May 1943’.
