# Supplementary material for: Surviving the Holocaust: Socio-demographic Differences Among Amsterdam Jews
Source: Eur J Popul. 2017 Jan 23;33(3):293–318. doi: 10.1007/s10680-016-9403-3 (PMC5493707; doi:10.1007/s10680-016-9403-3)
Supplement: Supplementary file 1 — Supplementary material 1 (DOCX 18 kb) [file 10680_2016_9403_MOESM1_ESM.docx]

Supplementary table 1: Estimates of odds ratios from logistic regression models for the association between socio-demographic characteristics and survival of the Holocaust including interaction terms.

|  |  | **Model 1** | | | **Model 2** | | |
| --- | --- | --- | --- | --- | --- | --- | --- |
|  |  | OR | 95% CI | P-value | OR | 95% CI | P-value |
|  | Intercept | 0.32 | 0.31, 0.34 | <0.001 | 0.38 | 0.36, 0,41 | <0.001 |
| *Gender (ref.=female)* | Male | 1.01 | 0.96, 1.06 | 0.623 | 0.75 | 0.70, 0.80 | <0.001 |
| *Age (ref.=15-30)* | 0-5 | 1.22 | 1.11, 1.33 | <0.001 | 1.05 | 0.94, 1.17 | 0.419 |
|  | 6-14 | 0.72 | 0.66, 0.79 | <0.001 | 0.68 | 0.62, 0.75 | <0.001 |
|  | 31-50 | 0.64 | 0.60, 0.67 | <0.001 | 0.51 | 0.48, 0.54 | <0.001 |
|  | 50+ | 0.37 | 0.35, 0.40 | <0.001 | 0.27 | 0.25, 0.29 | <0.001 |
| *Immigrant (ref.= born in NL)* | Born abroad | 1.56 | 1.36, 1.80 | <0.001 | 1.59 | 1.45, 1.75 | <0.001 |
| *Nationality (ref.=Dutch)* | German | 1.17 | 1.04, 1.31 | 0.008 | 1.15 | 1.03, 1.30 | 0.015 |
|  | Stateless | 1.27 | 1.09, 1.49 | 0.002 | 1.27 | 1.09, 1.49 | 0.002 |
|  | Polish | 1.45 | 1.17, 1.79 | 0.001 | 1.49 | 1.21, 1.84 | <0.001 |
|  | Other | 3.00 | 2.27, 3.95 | <0.001 | 3.01 | 2.29, 3.98 | <0.001 |
| *Religion (ref.=Dutch Israelite cong.)* | Secular Jews | 2.95 | 2.73, 3.18 | <0.001 | 2.95 | 2.73, 3.18 | <0.001 |
|  | Converted Jews | 6.07 | 4.67, 7.89 | <0.001 | 6.04 | 4.65, 7.85 | <0.001 |
|  | Portuguese Israelite congregation | 1.06 | 0.95, 1.18 | 0.288 | 1.06 | 0.95, 1.18 | 0.285 |
| *Family (ref.= married & children)* | Divorced, widowed & unmarried adults (18+) | 1.43 | 1.36, 1.51 | <0.001 | 1.52 | 1.44, 1.60 | <0.001 |
| *Mixed marriages* | Married to non-Jew | 5.35 | 5.01, 5.71 | <0.001 | 5.36 | 5.02, 5.72 | <0.001 |
| *Social class (ref.=no job)* | Higher managers & professionals | 1.94 | 1.68, 2.24 | <0.001 | 1.64 | 1.44, 1.87 | <0.001 |
|  | Lower manager, professional, clerical & sales | 1.14 | 1.08, 1.21 | <0.001 | 1.02 | 0.96, 1.08 | 0.495 |
|  | Foremen and skilled workers | 0.56 | 0.51, 0.61 | <0.001 | 0.52 | 0.47, 0.57 | <0.001 |
|  | Farm workers, farmers and fisherman | 0.60 | 0.28, 1.29 | 0.191 | 0.62 | 0.30, 1.27 | 0.189 |
|  | Lower-skilled workers | 0.54 | 0.50, 0.58 | <0.001 | 0.51 | 0.48, 0.55 | <0.001 |
|  | Unskilled workers | 0.45 | 0.40, 0.50 | <0.001 | 0.44 | 0.39, 0.49 | <0.001 |
|  | Unclassified | 3.07 | 2.08, 4.51 | <0.001 | 2.81 | 1.95, 4.07 | <0.001 |
| *Gender * Immigrant* | Male * Born abroad | 1.03 | 0.92, 1.15 | 0.611 |  |  |  |
| *Immigrant * Age* | Born abroad * 0-5 | 1.30 | 0.80, 2.12 | 0.289 |  |  |  |
|  | Born abroad * 6-14 | 1.50 | 1.20, 1.87 | <0.001 |  |  |  |
|  | Born abroad * 15-30 | 1.06 | 0.93, 1.20 | 0.459 |  |  |  |
|  | Born abroad * 50+ | 0.92 | 0.79, 1.07 | 0.256 |  |  |  |
| *Social class * Immigrant* | Higher managers etc. * Born abroad | 0.73 | 0.54, 1.00 | 0.051 |  |  |  |
|  | Lower manager etc. * Born abroad | 0.89 | 0.77, 1.03 | 0.113 |  |  |  |
|  | Foremen and skilled workers * Born abroad | 0.93 | 0.73, 1.18 | 0.526 |  |  |  |
|  | Farm workers etc. * Born abroad | 1.08 | 0.19, 6.06 | 0.930 |  |  |  |
|  | Lower-skilled workers * Born abroad | 1.21 | 1.01, 1,45 | 0.036 |  |  |  |
|  | Unskilled workers * Born abroad | 1.64 | 1.08, 2.48 | 0.020 |  |  |  |
|  | Unclassified * Born abroad | 0.94 | 0.32, 2.77 | 0.904 |  |  |  |
| *Gender * Age* | Male * 0-5 |  |  |  | 1.31 | 1.13, 1.52 | <0.001 |
|  | Male * 6-14 |  |  |  | 1.17 | 1.03, 1.34 | 0.016 |
|  | Male * 31-50 |  |  |  | 1.67 | 1.52, 1.83 | <0.001 |
|  | Male * 50+ |  |  |  | 1.78 | 1.62, 1.97 | <0.001 |
| Ref.=reference group. OR=odds ratio. CI= confidence interval. Adjusted for family relationship by taking into account clustering for household. | | | | | | | |
